# Optimized guide RNA structure for genome editing via Cas9

**DOI:** 10.18632/oncotarget.21607

**Published:** 2017-10-07

**Authors:** Jianyong Xu, Wei Lian, Yuning Jia, Lingyun Li, Zhong Huang

**Affiliations:** ^1^ Institute of Biological Therapy, Shenzhen University, Shenzhen, P.R. China; ^2^ Department of Pathogen Biology and Immunology, School of Medicine, Shenzhen University, Shenzhen, P.R. China

**Keywords:** RNA guided endonuclease, genome editing, Cas9, guide RNA, gRNA

## Abstract

The genome editing tool Cas9-gRNA (guide RNA) has been successfully applied in different cell types and organisms with high efficiency. However, more efforts need to be made to enhance both efficiency and specificity. In the current study, we optimized the guide RNA structure of *Streptococcus pyogenes* CRISPR (Clustered Regularly Interspaced Short Palindromic Repeats)/Cas (CRISPR-associated) system to improve its genome editing efficiency. Comparing with the original functional structure of guide RNA, which is composed of crRNA and tracrRNA, the widely used chimeric gRNA has shorter crRNA and tracrRNA sequence. The deleted RNA sequence could form extra loop structure, which might enhance the stability of the guide RNA structure and subsequently the genome editing efficiency. Thus the genome editing efficiency of different forms of guide RNA was tested. And we found that the chimeric structure of gRNA with original full length of crRNA and tracrRNA showed higher genome editing efficiency than the conventional chimeric structure or other types of gRNA we tested. Therefore our data here uncovered the new type of gRNA structure with higher genome editing efficiency.

## INTRODUCTION

Target genome editing is introducing the expected DNA changes into the specific site of the genome, producing cells lacking of a fragment of DNA sequence (knock-out), harboring extra DNA fragment (knock-in) or minimal DNA sequence alterations (target mutation/correction) [1–4]. This technology not only plays an important role in basic biology research for gene function studies but also holds a big promise for potential clinical applications in gene and cell therapy [1–4].

Taking the advantages of DNA repair process, engineered nucleases, such as ZFN (Zinc Finger Nucleases) and Talen (Transcription Activator-Like Effector Nucleases), induce double strand break or single strand nick to the specific site of the genomic DNA [5]. The DNA lesion would be repaired by homologous recombination (HR) in the presence of extra homologous DNA fragment or non-homologous end joining (NHEJ) which introduces small deletions, insertions or nucleotide alterations into the DNA [5]. Differing from ZFN and Talen, both of which are based on protein-DNA recognization, the new generation of engineered nuclease, RGEN (RNA Guided EndoNuclease) is based on base pairing between the gRNA (guide RNA) and target DNA [5]. It is much easier to construct, modify and attracts more attentions [1, 6, 7].

However, more efforts need to be made to enhance both efficiency and specificity of the RGEN [8]. Comparing with the original functional structure of guide RNA from *Streptococcus pyogenes* CRISPR (Clustered Regularly Interspaced Short Palindromic Repeats)/Cas (CRISPR-associated) system, which is composed of crRNA and tracrRNA, the widely used chimeric gRNA has shorter crRNA and tracrRNA sequence [9–12]. The deleted RNA sequence could form extra loop structure, which might enhance the stability of the guide RNA structure and subsequently the genome editing efficiency [9]. And in the current study we found that the chimeric structure of guide RNA with full length of original crRNA and tracrRNA showed higher genome editing efficiency than the conventional chimeric structure or other types of guide RNA we tested. Therefore our data here uncovered the new type of gRNA structure with higher genome editing efficiency.

## RESULTS

**Figure 1:**
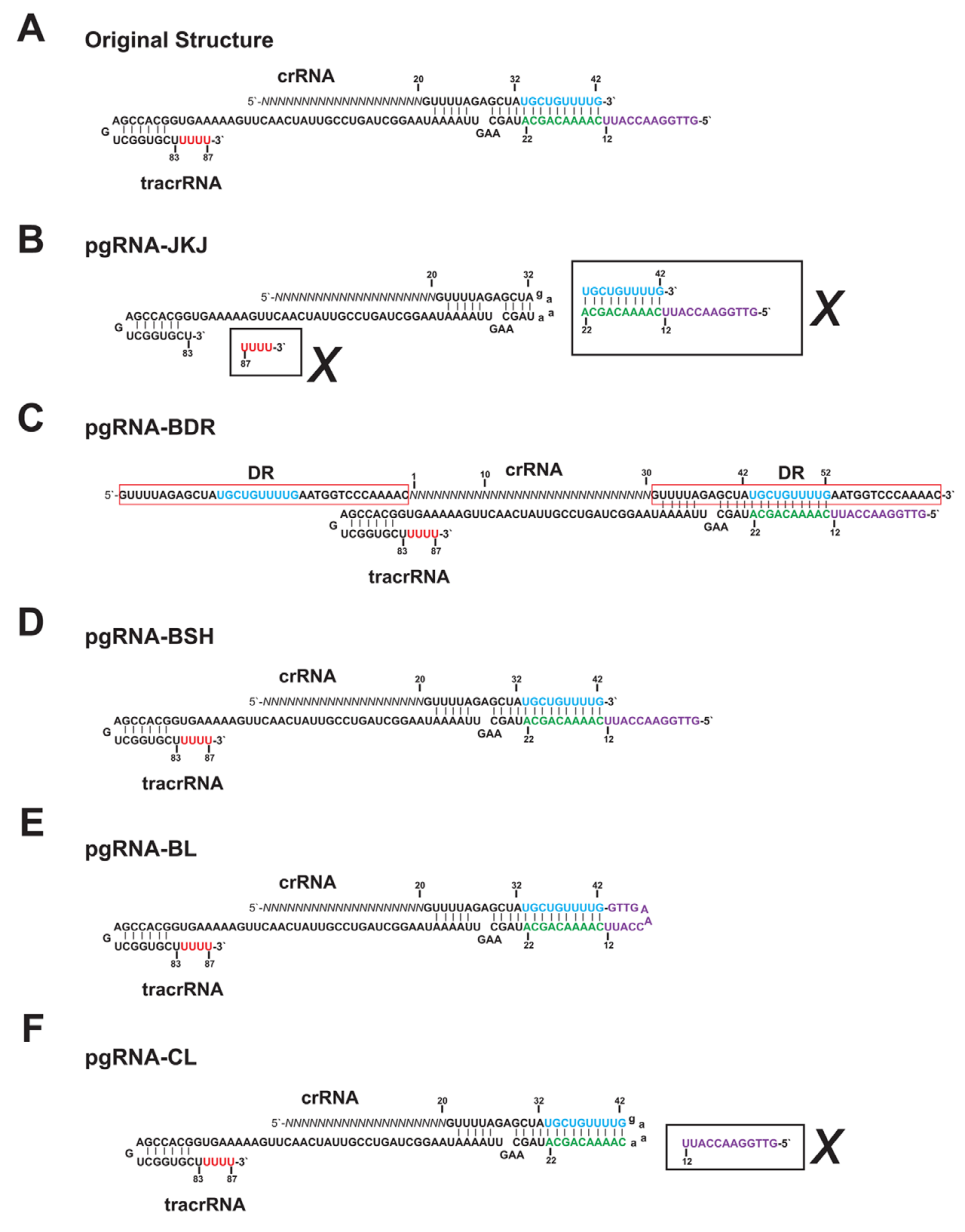
The sequence structures of the guide RNA **(A)** Original functional sequence structure of the guide RNA from *Streptococcus pyogenes* CRISPR/Cas system. **(B)** Sequence structure of pgRNA-JKJ. **(C)** Sequence structure of pgRNA-BDR. **(D)** Sequence structure of pgRNA-BSH. **(E)** Sequence structure of pgRNA-BL. **(F)** Sequence structure of pgRNA-CL. Sequences in the black box along with the cross mark indicate the sequences deleted from the full length of original gRNA sequence. Sequences in the red box indicate the DR (Direct Repeat) sequence. Sequences in blue and green indicate the region forming extra base pairing but deleted in the widely used gRNA structure gRNA-JKJ. Sequences in purple and red indicate the region not forming extra base pairing and deleted in the widely used gRNA structure gRNA-JKJ. Sequences in lowercase indicate the linker sequence.

Comparing with the original functional structure of guide RNA, which is composed of crRNA and tracrRNA, the widely used chimeric gRNA (pgRNA-JKJ) has shorter crRNA and tracrRNA sequence (Figure 1A, 1B). Furthermore, the deleted RNA sequence could form extra loop structure (Figure 1B), which might enhance the stability of the guide RNA structure and subsequently the genome editing efficiency. Thus the genome editing efficiency of five different forms of guide RNA was tested, including the widely used chimeric form (pgRNA-JKJ, Figure 1B), the native form reported before [10] (pgRNA-BDR, Figure 1C) and another three forms modified in the current study. Plasmid pgRNA-BSH was constructed by expressing the full length of original crRNA and tracrRNA individually (Figure 1D). Plasmid pgRNA-BL was constructed by expressing the full length of original crRNA and tracrRNA together directly (Figure 1E). Plasmid pgRNA-CL was constructed by replacing the unpaired part of tracrRNA of pgRNA-BL, which could not form base pairing with crRNA, with chimeric linker used for pgRNA-JKJ construction (Figure 1B, 1F).

Three different guide RNAs for human gene Desmin and four for human gene LAMP2, which are mutated genes in the hereditary cardiomyopathy [13, 14], were designed with web-based software ZiFiT Targeter. Corresponding modifications were made to adapt to guide RNA construction procedure (Supplementary Table 1).

**Figure 2:**
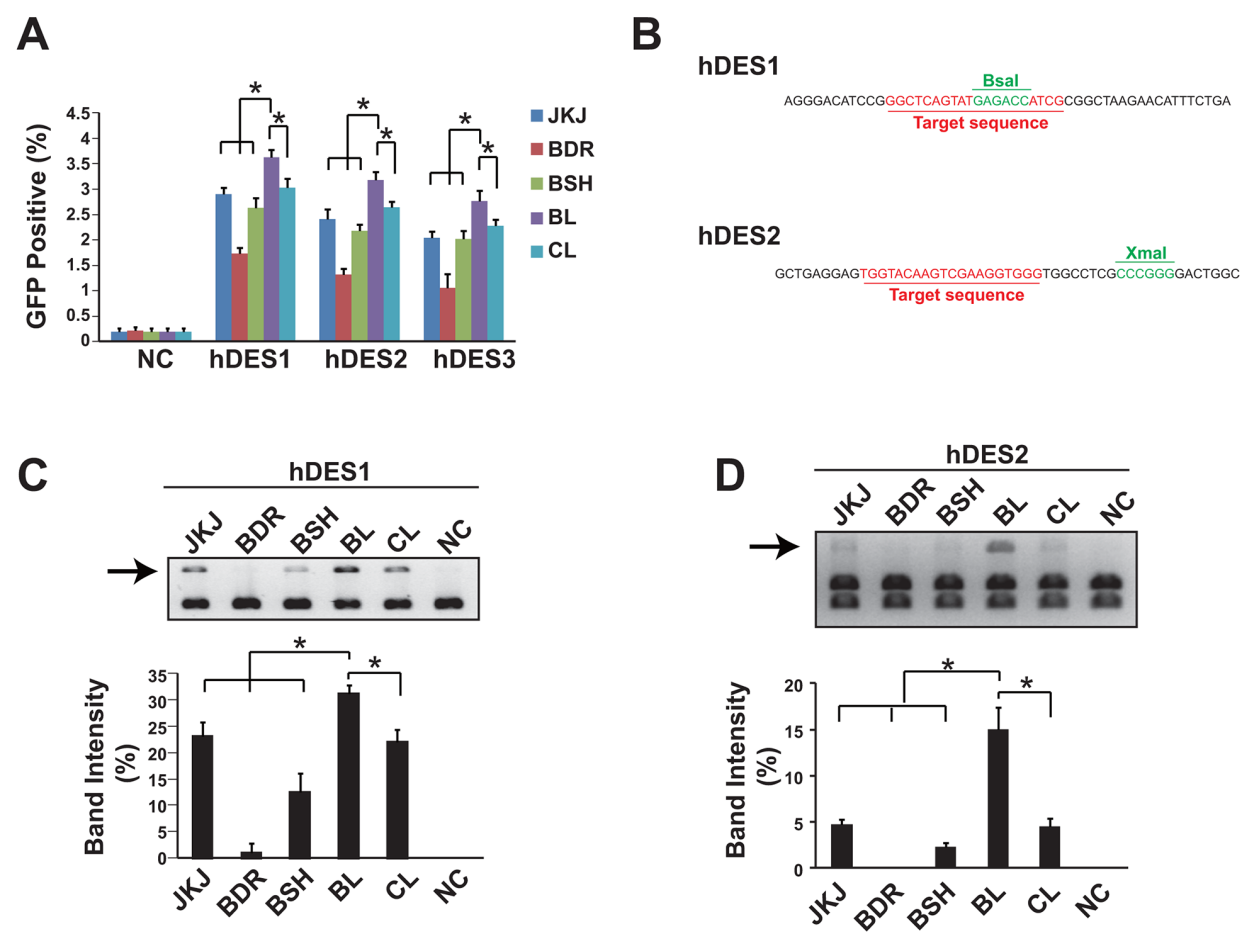
Genome editing efficiency comparison showed pgRNA-BL is the most efficient form of gRNA **(A)** Percentage of GFP positive cells via GFP reporter assay when cells transfected with different forms of gRNA plus Cas9. N=3. ^*^ indicates *P*<0.05. **(B)** The position of restriction enzyme sites and gRNA sites for hDES1 and hDES2. **(C, D)** NHEJ efficiency was measured by restriction enzyme site destruction assay for two sites on human gene Desmin. Up-panel showed representative figures of restriction enzyme digestion and gel electrophoresis; down-panel showed un-digested band density measured by Image J (n=3). ^*^ indicates *P*<0.05. NC: negative control; JKJ: pgRNA-JKJ; BDR: pgRNA-BDR; BSH: pgRNA-BSH; BL: pgRNA-BL; CL: pgRNA-CL; hDES1-3: three target sites on human gene Desmin.

A GFP reporter plasmid was applied to measure the genome editing efficiency of different forms of guide RNA. Data showed that the pgRNA-BL was the most efficient form among all five forms of gRNA tested in three sites of human gene Desmin, assessed in HEK293T cells (Figure 2A). As the GFP reporter (containing two 500bp length of overlapping GFP sequence and separated with the gRNA recognization sites) assay mostly measures the frequency of the homologous recombination, the NHEJ frequency was also evaluated. Two gRNA recognization sites of human gene Desmin were flanked by restriction enzyme sites (BsaI for gRNA site 1 and XmaI for gRNA site 2, Figure 2B). Once the NHEJ occurs, the restriction enzyme recognization sites would be eliminated in some DNA molecules and therefore could not be cut by the corresponding restriction enzymes. In accordance with the GFP reporter results, the pgRNA-BL was the most efficient form (Figure 2C, 2D). And this was further confirmed in the human gene LAMP2 (Supplementary Figure 1).

**Figure 3:**
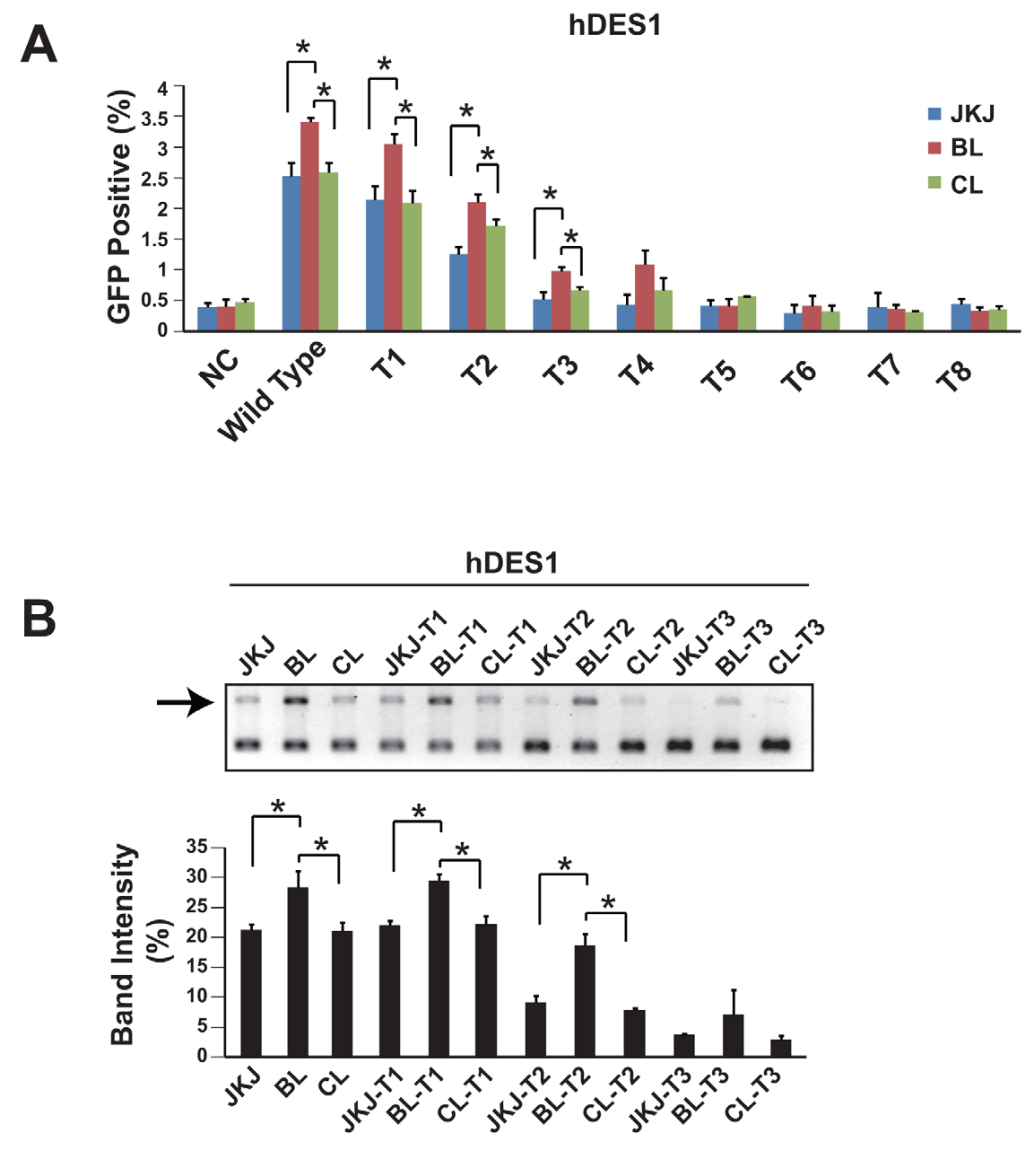
Genome editing efficiency comparison among pgRNA-JKJ, pgRNA-BL and pgRNA-CL with truncated gRNA **(A)** Percentage of GFP positive cells via GFP reporter assay when cells transfected with different forms of gRNA plus Cas9. N=3. ^*^ indicates *P*<0.05. **(B)** NHEJ efficiency was measured by restriction enzyme site destruction assay. Up-panel showed representative figures of restriction enzyme digestion and gel electrophoresis; down-panel showed un-digested band density measured by Image J (n=3). ^*^ indicates *P*<0.05. NC: negative control; Wild type: 20bp base pairing between gRNA and target DNA; T1: 19bp base pairing between gRNA and target DNA; T2: 18bp base pairing between gRNA and target DNA; T3: 17bp base pairing between gRNA and target DNA; T4-T8: 16bp to 12bp base pairing between gRNA and target DNA; JKJ: pgRNA-JKJ; BL: pgRNA-BL; CL: pgRNA-CL; hDES1: target site one on human gene Desmin.

It has been demonstrated that the truncated guide RNA, which has reduced number of base pairing between gRNA and target DNA sequence, could enhance the specificity [15]. Thus we also measured the genome editing efficiency of pgRNA-BL with truncated guide RNA. Data showed that during the guide RNA truncation, the genome editing efficiency of pgRNA-BL remained higher than pgRNA-CL and pgRNA-JKJ (Figure 3A, 3B). This was also confirmed in another human gene LAMP2 (Supplementary Figure 2). In addition to the HEK293T cell line, the genome editing efficiency comparison between pgRNA-BL and pgRNA-JKJ was also performed in Hela, SK-MES-1 and A549 cell lines (Supplementary Figure 3).

Thus, both the GFP reporter and NHEJ assays clearly indicated that the guide RNA structure of pgRNA-BL is much more effective than other structures we tested, including the widely used chimeric structure pgRNA-JKJ.

## DISCUSSION

The new generation of genome editing tool, RGEN, has paved a new way to manipulate the genome [1, 2]. The mechanism is similar to ZFN and Talen. All of them are based on two modules. One is used for binding to the target DNA sequence and other one for DNA cutting with endonuclease activity. The simple base pairing between the guided RNA of RGEN and the target DNA sequence confers an advantage over protein based DNA sequence recognization of ZFN and Talen. Comparing to the strategy of improving of the specificity of ZFN and Talen, the short and simple gRNA sequence (20bp for DNA pairing, 12-22bp for crRNA and 87bp for tracrRNA) is much easier to manipulate. The RGEN is a promising tool for high throughput genome manipulation, although more effects should be made to improve the efficiency and specificity [1, 4].

Comparing with the original functional structure of guide RNA, which is composed of crRNA and tracrRNA, the widely used chimeric gRNA has shorter crRNA and tracrRNA sequence [9–11]. The deleted RNA sequence could form extra loop structure, which might enhance the stability of the guide RNA structure and then the genome editing efficiency [9]. Thus we conducted the current research to compare different forms of guide RNA and we found that the chimeric structure of guide RNA with full length of original crRNA and tracrRNA showed higher genome editing efficiency than the conventional chimeric structure or other types of guide RNA we tested. Our results presented here are in accordance with previous reports that extending the crRNA and tracrRNA sequence would enhance the genome editing efficiency [16, 17].

The secondary structure of gRNA is crucial for Cas9 recognization and binding. And the base pairing between the crRNA and tracrRNA or inside of the crRNA/ tracrRNA is the basis of the gRNA secondary structure formation. The 20bp length of target DNA recognization sequence located in the 5 prime of the crRNA varies depending on the DNA region targeted. Thus the GC content or complexity of the 20bp sequence might affect the stability of the gRNA structure, as they also have the potential to form base pairing with the crRNA/ tracrRNA. The optimized structure of pgRNA-BL with extended crRNA and tracrRNA sequences would have more stabilized structure of crRNA and tracrRNA, therefore reducing the interfering effects of the 20bp sequence in the 5 prime of crRNA. Thus, the more gRNA with correct secondary structure, the more efficient genome editing occurs.

There are two potential applications. First, improving the genome editing efficiency with our optimized gRNA in the sites that the conventional gRNA shows low genome editing efficiency; second, improving the genome editing specificity with truncated target DNA recognization sequence located in the 5 prime of the crRNA. It has been demonstrated that the off-targets of Cas9 could be significantly reduced via using the truncated gRNA (shorter target DNA recognization sequence). However, the efficiency is normally also reduced with truncated gRNA [15, 18]. Thus, our modified gRNA, which has higher genome editing efficiency than the conventional structure, could remain the high genome editing efficiency while using the truncated gRNA, resulting in off-target reduction.

Therefore our data here uncovered the new type of gRNA structure with high genome editing efficiency. However, more efforts should be made to further enhance the efficiency and specificity. Furthermore, the mechanism of the secondary gRNA structure formation and maintenance needs more studies in detail.

## MATERIALS AND METHODS

### Plasmid construction

RGEN plasmids were obtained from Addgene, including FZ (Addgene 42229, pX260-U6-DR-BB-DR-Cbh-NLS-hSpCas9-NLS-H1-shorttracr-PGK-puro) [10], pgRNA-JKJ (Addgene 43860, MLM3636) [11], hCas9 (Addgene 41815, hCas9) [11]. Plasmid pgRNA-BDR was constructed by digesting the plasmid FZ with KpnI (New England Biolabs) and NotI (New England Biolabs), and deleting the gene Cas9. The resultant fragment was blunted and self-ligated with T4 ligase (New England Biolabs). Plasmid pgRNA-BSH was constructed by expressing the full length of original crRNA and tracrRNA under U6 and H1 promoter respectively. Plasmid pgRNA-BL was constructed by expressing the full length of original crRNA and tracrRNA together under U6 promoter. Plasmid pgRNA-CL was constructed by replacing the part of tracrRNA of pgRNA-BL, which could not form base pairing with crRNA, with chimeric linker used for pgRNA-JKJ construction. Details could be found in Figure 1.

Genome editing sites on human gene Desmin and LAMP2 were designed with ZiFiT Targeter (http://zifit.partners.org/ZiFiT/) [19]. The corresponding oligos were synthesized, mixed (5μL 100μM forward oligo, 5μL 100μM reverse oligo, 5μL 10 X NEB buffer 2 and 35μL ddH2O), annealed by heating up to 95 °C for 5 minutes and then gradually cooling down overnight in the water bath.

GFP reporter plasmid (pGFFP) was constructed as described before [20]. An extra LacZ gene flanked by EcoRV/XcmI sites was inserted into the reporter plasmid which would facilitate the clone process by applying blue-white screening. A 200bp DNA fragment harboring the RGEN target sites was PCR amplified and cloned into the GFP reporter plasmid with T-A clone method.

### Cell culture

The human cell line HEK293T, Hela, SK-MES-1 and A549 were obtained from the American Type Culture Collection (ATCC; Rockville, MD, USA) and cultured in DMEM (GIBCO, Shanghai, China) supplemented with 10 % FBS.

### GFP reporter assay

250ng of gRNA plasmid plus 250ng hCas9, 50ng GFP reporter plasmid and 1 μL Lipofactamine2000 (Thermo Scientific) were mixed to transfect 10x10^4^ HEK293T cells/well in p24 plate. The medium was refreshed daily and cells were analyzed 72 hours post transfection with Flow Cytometery (FC500, Beckman Coulter, Inc.).

### NHEJ (non-homologous end joining) measurement

500ng of gRNA plasmid plus 500ng hCas9 and 2 μL Lipofactamine2000 (Thermo Scientific) were used to transfect 20x10^4^ HEK293T cells/well in p12 plate. The medium was refreshed daily and DNA was extracted 72 hours post transfection with QIAamp DNA Blood Mini Kit (Qiagen). The RGEN target region was PCR amplified with AmpliTaq Gold 360 Master Mix (Thermo Scientific), column purified with illustra GFX PCR DNA and Gel Band Purification Kit (GE Healthcare Life Sciences) and digested with corresponding restriction enzymes according to the instructions (New England Biolabs). The digestion products were analyzed by 1.5% agarose gel electrophoresis and visualized by ultraviolet fluorescence (Gel Doc™ XR system, BIO-RAD) after staining with Novel Juice (Interchim).

### Statistical analysis

Data were analyzed by using SPSS software for Windows (SPSS Inc) and shown as means ± SEM (standard error of the mean). Student *t*-test was used for two-group comparison and one-way ANOVA for multiple group comparisons with normal data distribution, parametric test and Turkey post hoc tests. *P*≤0.05 was considered statistically significant.

## SUPPLEMENTARY MATERIALS FIGURES AND TABLE




