# Supplementary material for: Optimized guide RNA structure for genome editing via Cas9
Source: Oncotarget. 2017 Oct 7;8(55):94166–71. doi: 10.18632/oncotarget.21607 (PMC5706864; doi:10.18632/oncotarget.21607)
Supplement: Supplementary file 1 [file oncotarget-08-94166-s001.pdf]

Optimized guide RNA structure for genome editing via Cas9

SUPPLEMENTARY MATERIALS

Supplementary Table 1: Target sequences of the guide RNAs

See Supplementary File 1

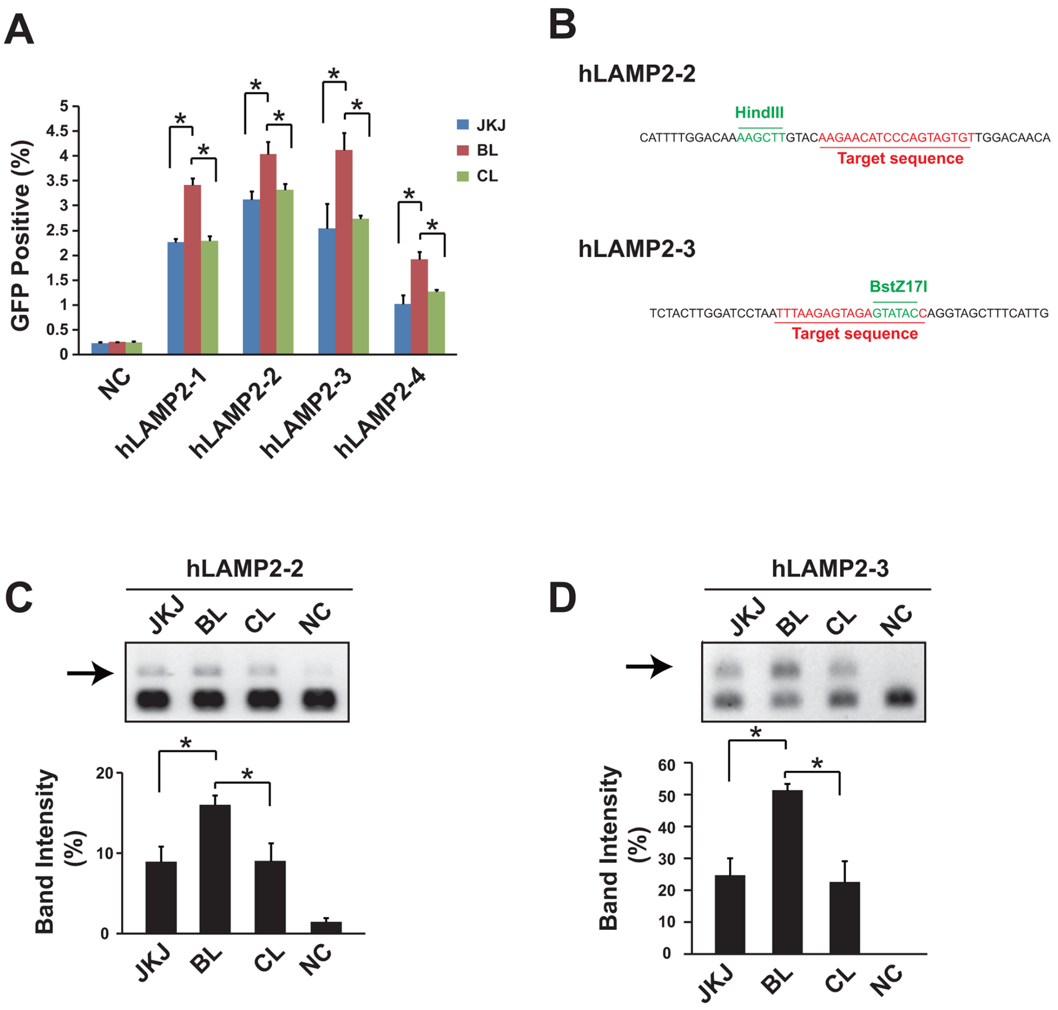

Supplementary Figure 1: Genome editing efficiency comparison showed pgRNA-BL is the most efficient form of gRNA. (A) Percentage of GFP positive cells via GFP reporter assay when cells transfected with different forms of gRNA plus Cas9. N=3. * indicates $P<0.05$. **(B)** The position of restriction enzyme sites and gRNA sites for LAMP2-2 and LAMP2-3. **(C, D)** NHEJ efficiency was measured by restriction enzyme site destruction assay for two sites on human gene LAMP2. Up-panel showed representative figures of restriction enzyme digestion and gel electrophoresis; down-panel showed un-digested band density measured by Image J (n=3). * indicates $P<0.05$. NC: negative control; JKJ: pgRNA-JKJ; BL: pgRNA-BL; CL: pgRNA-CL; hLAMP2-1, -2, -3 and -4: four different target sites on human gene LAMP2.

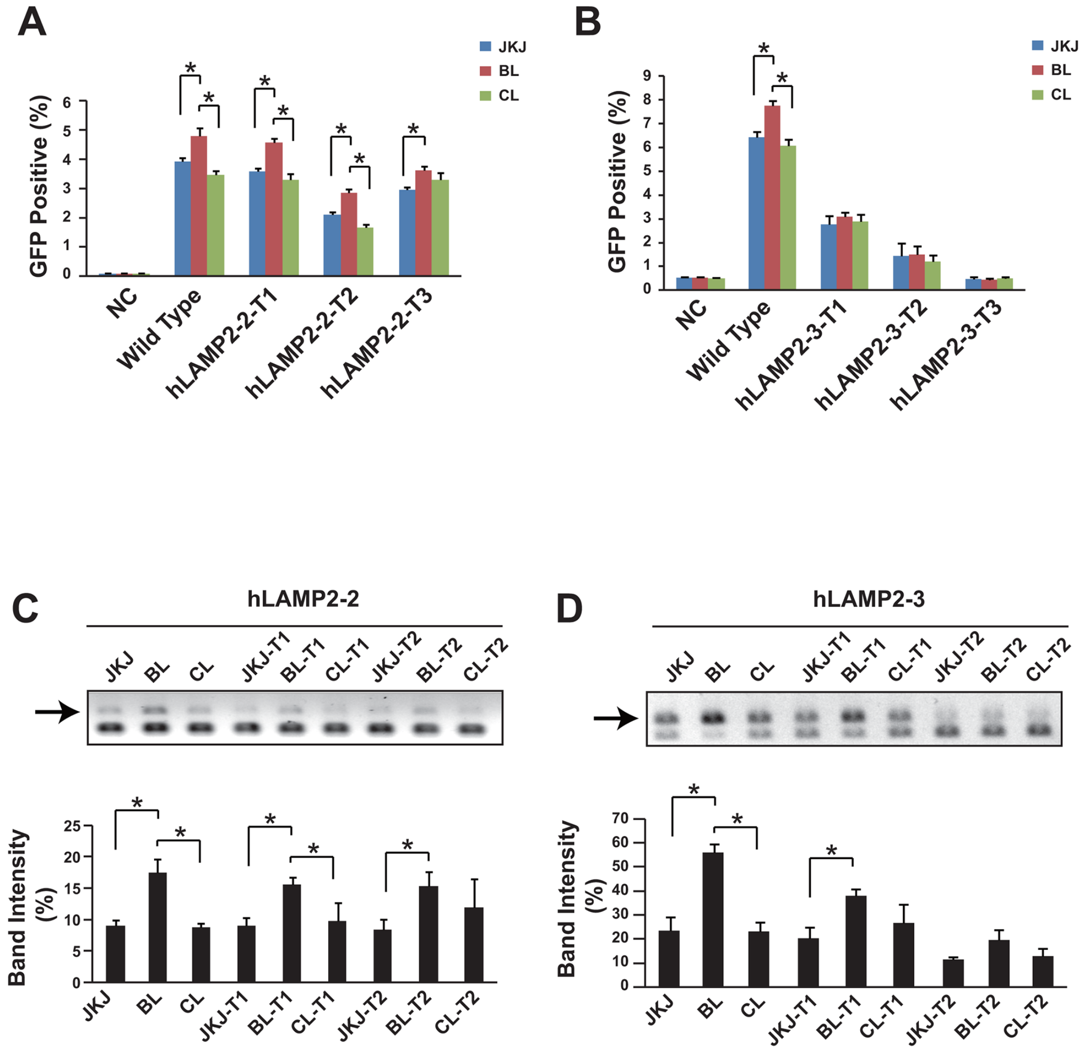

Supplementary Figure 2: Genome editing efficiency comparison with truncated gRNA. **(A, B)** Percentage of GFP positive cells via GFP reporter assay when cells transfected with different forms of gRNA plus Cas9. N=3. * indicates $P<0.05$. **(C, D)** NHEJ efficiency was measured by restriction enzyme site destruction assay. Up-panel showed representative figures of restriction enzyme digestion and gel electrophoresis; down-panel showed un-digested band density measured by Image J (n=3). * indicates $P<0.05$. NC: negative control; Wild type: 20bp base pairing between gRNA and target DNA; T1: 19bp base pairing between gRNA and target DNA; T2: 18bp base pairing between gRNA and target DNA; T3: 17bp base pairing between gRNA and target DNA; JKJ: pgRNA-JKJ; BL: pgRNA-BL; CL: pgRNA-CL; hLAMP2-2 and hLAMP2-3: two different target site on human gene LAMP2.

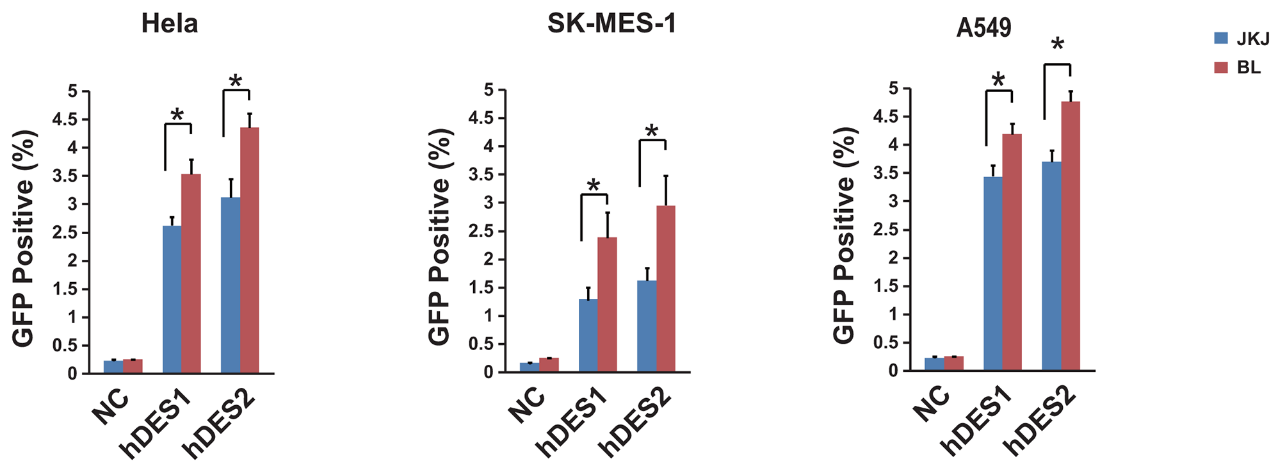

Supplementary Figure 3: Genome editing efficiency comparison in Hela, SK-MES-1 and A549 cell lines. Percentage of GFP positive cells via GFP reporter assay when cells transfected with different forms of gRNA plus Cas9. N=3. * indicates $P<0.05$. NC: negative control; JKJ: pgRNA-JKJ; BL: pgRNA-BL; hDES1 and hDES2: two target sites on human gene Desmin.
