# Supplementary material for: Optimized guide RNA structure for genome editing via Cas9
Source: Oncotarget. 2017 Oct 7;8(55):94166–71. doi: 10.18632/oncotarget.21607 (PMC5706864; doi:10.18632/oncotarget.21607)
Supplement: Supplementary file 2 [file oncotarget-08-94166-s002.docx]

| **Supplementary Table 1** | |  |
| --- | --- | --- |
|  | Oligo Name | Target Sequence (5`--3`) |
|  | hDESgRNA1 | GGCTCAGTATGAGACCATCG |
|  | hDESgRNA2 | TGGTACAAGTCGAAGGTGGG |
|  | hDESgRNA3 | GCACATCCAAGCTGGGATTC |
|  | hLAMP2gRNA1 | TGATGTTGTCCAACACTAC |
|  | hLAMP2gRNA2 | AAGAACATCCCAGTAGTGT |
|  | hLAMP2gRNA3 | TTTAAGAGTAGAGTATACC |
|  | hLAMP2gRNA4 | TACAAGCTTTTGTCCAAAA |
|  |  |  |
|  | hDESgRNA1-T1 | GCTCAGTATGAGACCATCG |
|  | hDESgRNA1-T2 | CTCAGTATGAGACCATCG |
|  | hDESgRNA1-T3 | TCAGTATGAGACCATCG |
|  | hDESgRNA1-T4 | CAGTATGAGACCATCG |
|  | hDESgRNA1-T5 | AGTATGAGACCATCG |
|  | hDESgRNA1-T6 | GTATGAGACCATCG |
|  | hDESgRNA1-T7 | TATGAGACCATCG |
|  | hDESgRNA1-T8 | ATGAGACCATCG |
|  |  |  |
|  | hLAMP2gRNA2-T1 | AGAACATCCCAGTAGTGT |
|  | hLAMP2gRNA2-T2 | GAACATCCCAGTAGTGT |
|  | hLAMP2gRNA2-T3 | AACATCCCAGTAGTGT |
|  |  |  |
|  | hLAMP2gRNA3-T1 | TTAAGAGTAGAGTATACC |
|  | hLAMP2gRNA3-T2 | TAAGAGTAGAGTATACC |
|  | hLAMP2gRNA3-T3 | AAGAGTAGAGTATACC |
|  |  |  |
